# Feasibility of Using Accelerometer Measurements to Assess Habitual Physical Activity in Rural Heart Failure Patients

**DOI:** 10.3390/geriatrics2030023

**Published:** 2017-07-14

**Authors:** Lufei Young, Melody Hertzog, Susan Barnason

**Affiliations:** 1 College of Nursing, Augusta University, Augusta, GA 30912, USA; 2 College of Nursing, University of Nebraska Medical Center, Omaha, NE 68105, USA; mhertzog@unmc.edu (M.H.); sbarnaso@unmc.edu (S.B.)

**Keywords:** ActiGraph, accelerometer, feasibility, reliability, physical activity, heart failure, rural health

## Abstract

(1) Background: Physical inactivity is prevalent in rural heart failure (HF) patients. To evaluate the effectiveness of interventions aimed at improving physical activity (PA), we need an accurate, reliable PA assessment tool that is feasible and acceptable to HF patients. The purpose of this study was to examine the feasibility and reliability of using an accelerometer to assess HF patients’ PA. (2) Method: A total of 100 HF patients discharged from a rural hospital participated in the study and wore an accelerometer at baseline, 3, and 6 months following discharge. (3) Result: The daily average wear time across all three time points was 15.7 (±3.3) h for weekdays, and 15.8 (±3.7) h for weekends. Approximately 50% of the participants adhered to the device wear protocol at baseline, 3, and 6 months. Factors related to wear time were also examined. Acceptable reliability assessed by intra-class correlation (ICC > 0.879) was found for daily activity calories, activity counts per minutes, and time spent on moderate or greater PA. (4) Conclusion: The present findings suggest that an accelerometer is a feasible and reliable measure of habitual PA in rural HF patients over time.

## 1. Introduction

Heart failure (HF) is a complex clinical syndrome caused by structural and/or functional impairment of cardiac ventricular filling or output [1]. Based on left ventricular ejection fraction values (above 50% vs. below 40%) obtained from echocardiograph exams, HF can be further classified as HF with preserved (HFpEF) or reduced ejection fraction (HFrEF) [2]. Exercise intolerance is a primary symptom in both HFpEF and HFrEF patients [3,4]. On the other hand, HF patients who are physically active have lower risk of adverse outcomes (e.g., mortality, complications), as well as improved functioning and independence [5,6]. To achieve significant improvement in clinical and functional outcomes, it is recommended that HF patients engage in at least 150 min per week of moderate exercise (30 min a day, five times a week) or 75 min per week of vigorous exercise (or a combination of moderate and vigorous activity), in addition to 2 days per week of muscle strengthening [7]. In spite of the definitive benefits, evidence shows a higher prevalence of sedentary behavior in rural older adults [8,9]. Specifically, individuals with HF have been found to be less physically active than their urban counterparts [10,11,12,13].

To develop and evaluate interventions to improve physical activity (PA), an accurate, reliable, and feasible PA assessment tool is needed for this population. To date, PA in HF patients has been frequently measured using questionnaires [14,15]. Self-reported PA measures are, however, often inaccurate and unreliable due to frequent pathophysiological changes and cognitive impairment in the elderly population [16]. Inaccurate data make it difficult to examine the correlates of PA, evaluate intervention effects, and monitor changes in PA over time. In contrast, objective measures, such as accelerometers, are believed to offer more accuracy without recall and response bias compared to self-reported measures [17,18,19,20,21,22,23]. The accelerometer is the most frequently used objective indicator of daily PA and calorie expenditure in research and clinical settings [18,24]. However, adherence to the monitor wear requirement can be challenging for HF patients. Due to compromised cardiac function, HF patients often have poor physical tolerance [3], and wearing a monitoring device could be burdensome and interruptive to their daily routine. Moreover, sufficient wear time is required to obtain a reliable measure of habitual PA [25]. To obtain a reliable estimation of habitual PA, a minimum of eight hours monitor-wear per day over seven days is required [26]. Whether HF patients can tolerate long hours of monitor wear has not been reported, nor has the reliability of using the accelerometer to assess habitual PA been reported. The development and evaluation of interventions to improve HF patients’ PA is warranted, which necessitates the use of a feasible and reliable assessment tool to monitor PA in this population. Therefore, to fill the knowledge gap, we conducted a study aimed at assessing the feasibility and reliability of using an accelerometer to measure habitual PA levels in HF patients over time. The factors influencing device wear time duration were also examined.

## 2. Materials and Methods

### 2.1. Research Design

This study was a secondary analysis using the data from a randomized controlled trial aimed at examining the effectiveness of a 12-week home-based intervention to improve HF self-management adherence [10]. In addition to PA, the original study evaluated the impact of an intervention on guideline adherence for other self-management activities (e.g., daily weighing, taking prescribed medications, following a low-salt diet, and keeping scheduled doctor appointments) [10]. The original study is registered on the Clinical Trial website (NCT01964053) and was conducted between September 2013 and October 2015. The study protocol was approved by the University Institutional Review Board and the rural hospital ethics committee. All participants gave written informed consent [10]. Taking a different focus from the original study, the purpose of this secondary analysis was to determine the feasibility (measured by wear time and patients’ acceptability) and reliability of using an accelerometer to assess habitual PA in HF patients over time.

### 2.2. Sample and Setting

Patients were recruited from a rural critical access hospital, and eligible participants: (1) were aged 21 years or older; (2) had HF as one of their discharge diagnoses; (3) were classified as New York Heart Association (NYHA) class I and had at least one HF-related hospitalization or emergency department visit in the previous year; (4) were discharged to home; (5) passed a mini-cog screen test [27]; (6) understood English; and (7) had access to a phone. We excluded patients who: (1) had depressive symptoms, received a score of 3 or above on the Patient Health Questionnaire-2 (PHQ-2) [28]; (2) were diagnosed with liver cirrhosis; (3) were diagnosed with chronic renal failure; or (4) were diagnosed with other end stage and/or terminal illness (e.g., cancer) which limited the patient’s ability to perform moderate or above PA. The study setting was described in more detail in a previously published manuscript [10].

### 2.3. Measures and Procedure

Data were collected from both intervention and control groups at three time points: baseline (during the first week after hospital discharge), and at 3 and 6 months after baseline. Participants wore an Actigraph GT3X-BT accelerometer (ActiGraph LLC, Pensacola, FL, USA) attached to an elastic belt around the waist and positioned on the non-dominant hip [29,30] for a minimum of 8 h a day on 7 consecutive days, which is the most commonly reported wearing time period [31,32]. According to manufacture specifications, the accelerometer was initiated through ActiLife v6 (ActiGraph LLC, Pensacola, FL, USA) at a sampling rate of 60 Hz [33]. ActiLife digital filtering algorithm, the “normal filter”, was used to minimize acceleration noise outside the normal human activity frequency bandwidth [34]. A 60-minute or greater duration of zero activity counts were defined as non-wear time and excluded from the analyses [30]. The Freedson algorithm [35] was used to quantify wake and sleep periods. After being downloaded in ActiLife software, data were converted into vector magnitude (VM) counts at 60-s epochs. The Freedson Combination (1998) energy expenditure formula was used to compute daily activity calories, and the Freedson Adult (1998) Cut Point classification was used to determine daily minutes spent on sedentary, light, moderate, vigorous, and very vigorous PA [36]. The cut point protocol has been validated in multiple studies [30,33,34,37]: (1) ≤99 counts per minutes (CPM; sedentary); (2) 100–1951 CPM (light PA); (3) 1952–5724 CPM (moderate PA); (4) 5725–9498 CPM (vigorous PA); and (5) ≥9499 CPM (very vigorous).

To assess the feasibility of using an accelerometer to evaluate habitual PA over time, we first calculated average daily accelerometer wear time and the percentage of participants who wore the device for 7 days at each data collection point. Besides PA data, participants’ sociodemographic and clinical data were collected to describe sample characteristics. Based on findings in other studies [13,38], we collected and analyzed the following data at three time points to identify factors related to monitor wear time, including working status, medications, healthcare utilization (i.e., hospitalizations), and other clinical data relevant to PA and functioning. To assess patients’ acceptability, we conducted individual and focus group interviews asking participants to share their experience with wearing the accelerometer at each follow-up time point (e.g., perceived burden, challenges and barriers with wearing the accelerometer in accordance with the protocol). Interview data were transcribed verbatim and analysis was conducted using NVivo 10 (QSR International, Burlington, MA, USA, 2012). Each transcript was coded using an inductive process [39]. A second researcher reviewed the data to confirm the coding. Discrepancies in coding between researchers were discussed until consensus was achieved.

### 2.4. Statistical Analysis

All quantitative analyses were performed using IBM SPSS 23 (IBM SPSS Statistics for Windows, IBM Corp., Armonk, NY, USA). A *p*-value less than 0.05 indicated statistical significance. We excluded participants who did not have at least one valid day of wear (a minimum of 8 h of wear time) at each time point. We reported means and standard deviations for continuous variables and frequencies and percentages for categorical variables. The average accelerometer wear time per day was first computed for each person across their valid days, and then a group mean was calculated across all individuals to generate an unweighted mean. The average wear time in hours was computed for both weekdays and weekends at each time point (baseline, 3, and 6 months).

The factors associated with wear time duration were identified using linear mixed-effects longitudinal regression models. The models included random effects to account for dependence within individuals and a first-order autoregressive (AR) covariance structure to account for repeated observations per participant over time. To account for the impact of multiple monitor wears on the outcome, logistic mixed-effects longitudinal regression models were constructed using the same sets of fixed and random effects and AR covariance structures.

Two-way random models of intra-class correlations (ICCs) were used to establish the reliability of a single day accounting for the between-day and inter-person variability [25]. The monitor reliability assessed by ICCs was investigated for the following variables: daily activity calories in Kcal, VM activity counts per minutes, and minutes spent on moderate or greater intensity PA. The reliability was considered substantial if the ICC ranged from 0.8 to 1.0 [40].

## 3. Results

### 3.1. Sample Characteristics

A total of 105 participants were enrolled in the study. The final sample used for analysis included 100 participants (64 females and 36 males). Participants’ summary demographic and clinical characteristics are presented in Table 1. Participants’ mean age was 70.2 (±12.21) years. The participants tended to be female (64%), white (95%), retired (71%), and have an average of 12.9 (±2.3) years of education. Most participants’ cardiac functioning was classified at New York Heart Association (NYHA) level II (49%) or III (42%), with a preserved ejection fraction (55.7 ± 11.1). Participants had an average body mass index (BMI) of 32.3 ± 7.1 (56% obese and 28% overweight). The average number of comorbidities at baseline was 8 (±2.6), including hypertension (99%), coronary artery disease (94%), arthritis or degenerative joint disease (89%), and hypercholesterolemia (84%). Participants reported taking an average of 16.2 (±8.8) pills per day. There were no significant differences between intervention and control group for all demographic and clinical variables (Table 1).

### 3.2. Feasibility

Of the participants who met accelerometer wear time criteria, the average wear time per day across all three time points was 15.7 (±3.3) h for weekdays and 15.8 (±3.7) h for weekends. Table 2 presents the average wear time for both weekdays and weekends at each time point. Average differences between weekend and weekday wear hours were 35 min or less; however, it is worth noting that there were fewer participants with valid wear days on weekends than on weekdays.

Participants were asked to wear the Actigraph for 7 consecutive days, at least 8 h per day. At the three time points, respectively, 8%, 2%, and 5% of participants did not have any valid wear days. Among those who wore the accelerometer for at least one day, the average number of days wearing the accelerometer was 5.9 (±1.7) days, 6.2 (±1.4) days, and 5.9 (±1.4) days, respectively. Of the 100 participants at baseline, 54% achieved a 100% compliance rate. A total of 56% and 45% of participants achieved this at 3 and 6 months, respectively. In spite of the low absolute compliance rates, Table 3 shows that a large percentage of participants wore the Actigraph for at least 5 days at each time point.

### 3.3. Reliability

To assess the reliability of the accelerometer in evaluating habitual PA in rural HF patients, we computed single day intraclass correlations (ICC) values for three PA variables as described earlier. ICC values for 7-day mean activity calories, counts per minute, and minutes in moderate or greater intensity activity (Table 4) were all greater than 0.8, ranging from 0.879 to 0.966 at three follow up time points. This suggested that the typically recommended 7 days of accelerometer wear yielded acceptably reliable estimates of habitual PA in HF patients over time.

### 3.4. Factors Associated with Monitor Wear Time

The significant variables included in the final model were age (*r* = −0.25, *p* = 0.016), log transformed brain natriuretic peptide (*r* = −0.27, *p* = 0.010), insulin users (*F* = −2.8126, *p* = 0.007) at baseline, and being re-hospitalized (*F* = 2.6832, *p* = 0.023) at 3 months.

### 3.5. Acceptance

Participants’ acceptance of the accelerometer was assessed using interview data. Several common challenges and barriers were evident. First, it was commonly perceived as difficult to wear the monitor during holidays or unexpected events (e.g., being admitted to the hospital, acute illness, traveling). Participants reported discomfort and problems while wearing the monitor, including (1) skin problems (e.g., causing perspiration, trapping moisture, rash and pruritus); (2) fit issues (e.g., too tight or too loose); (3) interference with other self-care activities (e.g., shower, dressing, toileting); (4) interference with scheduled medication; and (5) difficulty with putting it on and/or taking it off. Several participants mistakenly thought the monitor was an alert system (e.g., Lifeline). One participant called 911 when she noticed the blinking light. Participants doing aquatic exercise were disappointed with not being able to wear the monitor in water. Some participants commented that the pre-addressed and pre-paid envelope and reminder calls from the research assistants were helpful, reducing stress and burden. Most participants reported that their positive relationship with the research investigator increased their willingness to wear the device.

## 4. Discussion

Identifying a feasible and reliable PA assessment tool accepted by HF patients is critical [18]. Without accurate and appropriate measurement, it would be challenging to design interventions to promote PA and evaluate their effects. The most common measure of PA in the HF population is a self-reported questionnaire. However, frequent physiological change and cognitive decline increase the difficulty of obtaining accurate PA measurement through self-reported questionnaires in older adults with HF [18]. In contrast, accelerometers are gaining in popularity because of their accuracy in examining PA levels in older adults with multiple chronic conditions. However, to the best of the authors’ knowledge, the feasibility, reliability, and patient-reported experience with using an accelerometer to assess PA in rural HF patients have not been reported elsewhere. Our study may help fill this knowledge gap.

Consistent with evidence reported by Garata [32], the accelerometer proved to be a highly reliable tool to assess PA level in rural HF patients. In addition to reliability, we examined the feasibility of using an accelerometer to assess habitual PA level in HF patients. One feasibility indicator is compliance to the accelerometer wear protocol, which has not been reported in HF patients. In our study, HF participants’ compliance with wear time protocol (45–56%) was similar to that found in the general population [41]. In addition, the daily average wear time was comparable to that found in other patient populations (e.g., patients with mental illness [41] or traumatic brain injury [42]), and longer than Chronic Obstructive Pulmonary Disease (COPD) patients (15 vs. 10 h per day) [31].

To date, there have been no known reports regarding factors related to compliance with wear time protocol in HF patients. Contrary to Lee’s finding in the general population [43], older participants in our study were less likely to comply with the wear time protocol. Factors influencing wear time included age, insulin use, being hospitalized, and B-type natriuretic peptide (BNP) value. BNP is a gold standard test of chronic HF exacerbation. Participants who were older and had increased BNP had less daily wear time. It is postulated that participants who were younger and without HF exacerbation were more capable of tolerating longer hours of wear time and felt less study burden. Using insulin at baseline was significantly associated with greater average daily wear time. Patients taking insulin might have been used to the complex and rigid self-management tasks in terms of meal planning and drug administration schedules [44], and therefore might be more likely to follow the wear time protocol [44]. Moreover, being re-hospitalized at 3 months was associated with shorter daily monitor wear time. This finding is congruent with participant-reported data recorded in our field notes; participants expressed the burden and inconvenience of wearing the monitor when they were re-hospitalized. Similar to previous studies [41], we found that strategies for improving compliance with the accelerometer wear protocol include a daily log, reminder calls, and incentives (e.g., gift cards or cash). In addition, we found that a positive relationship between the participant and investigator plays a critical role in improving and sustaining compliance with the monitor wear protocol.

Several limitations exist in this study. First, the study was conducted in one rural critical access hospital using a convenience sample with voluntary participation, which has the limitation of a potential participant selection bias. For instance, patients who were habitually inactive might have chosen not to participate the study. Therefore, caution should be used in generalizing the results to rural HF patients. Secondly, the waist worn accelerometer was used in our study. The feasibility results may not be the same if other types of objective measures (e.g., wrist worn or thigh worn accelerometers) were used. Lastly, this is a secondary analysis using data from the original study that was not designed to test the feasibility and reliability of an accelerometer for measuring habitual PA in HF patients. Therefore, to identify the optimal PA measures for HF patients, more qualitative and quantitative research is needed to validate our findings and help gain a better understanding of patient perspectives and other factors influencing adherence to a monitor wear protocol (e.g., medication intakes).

### Contributions and Implications

The study has some important strengths. First, this is the first study to examine the feasibility, reliability, and acceptance of using an accelerometer to assess habitual PA in rural HF patients. Evidence has shown that rural HF patients have greater challenges staying physically active [10,12,13], supporting the need for targeted interventions to promote PA in this underserved population. However, without a reliable and feasible measure, it would be difficult to determine the intervention effects over time. Second, the data were collected across multiple time points, supporting the utility of accelerometers in longitudinal studies to assess habitual PA pattern and intervention effects. Last, the findings of this study have implications for future research in the field of PA assessment. The information will be valuable in developing recruitment strategies, specifying participant instructions about length of time and proper wearing of the monitor, utilizing an activity log-book, identifying risk factors of non-compliance, and developing strategies to reduce deviation from protocols.

## 5. Conclusions

Pathological and non-pathological aging creates unique challenges to accurately assess HF patients’ habitual PA over time. Selecting the appropriate tool to assess PA for HF patients is difficult and complex. It would be impossible to examine intervention effects if patients did not comply with the monitor wear protocol. In this study, we concluded that the accelerometer is a reliable and feasible measure of habitual PA level in rural HF patients. More studies are needed to confirm our findings and promote the use of accelerometer to predict HF exacerbation and detect intervention effects in older adults living with disabling conditions.

## Figures and Tables

**Table 1:** Participants’ demographic and clinical characteristics

Variables	All (*n* = 100 )	Intervention Group (*n* = 51)	Control Group (*n* = 49)
Demographic data			
Age (years)	70.2 ± 12.2	68.7 ± 11.8	71.8 ± 12.6
Male	36 (36)	24 (47.1)	12 (24.5)
Education (years)	12.9 ± 2.3	13 ± 2.4	12.8 ± 2.1
Caucasian	95 (95)	48 (94.1)	47 (95.9)
Married/living with partner	50 (50)	31 (60.8)	19 (38.8)
Currently employed outside home	29 (29)	16 (30.8)	13 (26.5)
Annual family income (<$30,000)	51 (51)	24 (47.10)	27 (55.1)
Risk factor profile			
Body mass index (kg/m²)	32.3 ± 7.1	33.4 ± 7.4	31.2 ± 6.8
Clinical data			
Number of comorbidities	8 ± 2.6	7.8 ± 2.5	8.0 ± 2.7
Hypertension	99 (99)	51 (100.0)	48 (98.0)
Coronary artery disease	94 (94)	46 (90.2)	48 (98)
Arthritis degenerative joint disease	89 (89)	43 (84.3)	44 (89.8)
Hypercholesterolemia	84 (84)	43 (84.3)	41 (83.7)
Diabetes mellitus with or without complications	41 (41)	41 (80.4)	33 (67.4)
Dyspepsia	50 (50)	24 (47.1)	26 (53.1)
Peripheral vascular disease or lower extremity edema	45 (45)	22 (43.1)	23 (46.9)
Chronic obstructive pulmonary disease	38 (38)	22 (43.1)	16 (32.7)
Chronic renal disease	23 (23)	12 (23.5)	11 (22.4)
Number of medications taking per day	16.2 (±8.8)	16.4 ± 10.0	15.9 ± 7.4
Cardiac function			
Functional class (NYHA)			
II	49 (49)	15 (29.4)	34 (69.4)
III	42 (42)	29 (56.9)	13 (26.5)
Ejection fraction ^a^	55.7 ± 11.1	53.4 ± 12.9	58.3 ± 8.1
Ejection fraction <50% ^a^	16 (16)	12 (23.5)	4 (8.2)

^a^ Ejection fraction was available for *n* = 47 in the intervention group, *n* = 41 in the usual care group. Percentages include cases with missing data in the denominator. NYHA = New York Heart Association.

**Table 2:** Average wear time in hours for both weekdays and weekends at each time point.

Time Point	Overall Mean (±SD)	Weekdays Mean (±SD)	*n*	Weekends Mean (±SD)	*n*
Baseline	15.4 (±3.6)	15.4 (±3.8)	92	16.0 (±3.8)	81
3 months	16.1 (±3.2)	16.1 (±3.1)	98	16.3 (±3.8)	90
6 months	15.4 (±3.1)	15.6 (±3.1)	95	15.2 (±3.4)	84

**Table 3:** Proportion of participants wearing more than 5, 6, and 7 days over time.

Time Point	7 Days	≥6 Days	≥5 Days
Baseline	54%	69%	76%
Month 3	56%	80%	87%
Month 6	45%	65%	82%

**Table 4:** Intraclass correlations (ICC) calculated at three time points *.

Variables	Baseline	3 Months	6 Months
*n*	54	57	45
Activity calories	0.966	0.940	0.955
Activity counts (vector magnitude)	0.964	0.939	0.937
Minutes in moderate intensity or above PA	0.914	0.912	0.879

* using participants who wore the Actigraph for 7 days at that time point.
